# Incidencia de dengue y su relación con el índice oceánico de El Niño, como variable sensible para anticipar brotes en la región Caribe colombiana

**DOI:** 10.7705/biomedica.7933

**Published:** 2025-11-27

**Authors:** Alexander Salazar-Ceballos, Lídice Álvarez-Miño

**Affiliations:** 1 Grupo de Investigación en Salud - GRISAL, Programa de Enfermería, Universidad Cooperativa de Colombia, Santa Marta, Colombia Universidad Cooperativa de Colombia Universidad Cooperativa de Colombia Santa Marta Colombia; 2 Grupo de Investigación Ciencias del Cuidado Enfermería - GICCE, Programa de Enfermería, Universidad del Magdalena, Santa Marta, Colombia Universidad del Magdalena Universidad del Magdalena Santa Marta Colombia

**Keywords:** dengue, clima, cambio climático, región del Caribe, El Niño oscilación del sur, estudios de series de tiempo, alerta temprana, dengue, climate, climate change, Caribbean region, El Niño-Southern Oscillation, time series studies, early warning

## Abstract

**Introducción.:**

El informe *Lancet Countdown 2023* para Latinoamérica indica que el aumento de las temperaturas influye en la transmisión del virus del dengue. En la región Caribe de Colombia, se ha identificado una asociación significativa entre la incidencia de dengue y variables climáticas, como la temperatura, la humedad y la precipitación.

**Objetivo.:**

Analizar la relación entre la tasa de incidencia de dengue y el índice oceánico de El Niño en los departamentos de la región Caribe colombiana entre el 2021 y el 2023.

**Materiales y métodos.:**

Se llevó a cabo un estudio ecológico de serie de tiempo, utilizando modelos de regresión no lineal con desfase y modelos autorregresivos integrados de media móvil en los siete departamentos de la región Caribe. Para los análisis descriptivos y los modelos autorregresivos, se emplearon los programas JASP y RStudio. Para los análisis no lineales y con desfase, se usó el paquete dlnm de RStudio.

**Resultados.:**

Se encontró una relación positiva y significativa entre el índice oceánico de El Niño y la tasa de incidencia de dengue en el 2023, año en el que se presentó el fenómeno de El Niño. Los departamentos de Bolívar, Cesar, Córdoba y Magdalena tuvieron correlaciones positivas. También, se observó una relación no lineal entre El Niño o La Niña y la incidencia de dengue, con un mayor impacto durante la fase de El Niño.

**Conclusiones.:**

El índice oceánico de El Niño se presenta como un indicador climático útil para monitorear el aumento de casos de dengue en los departamentos analizados de la región Caribe colombiana.

## Abstract (en)

**Introduction.:**

The *Lancet Countdown 2023* report for Latin America indicates that rising temperatures influence the transmission of the dengue virus. In Colombia’s Caribbean region, a significant association has been identified between dengue incidence and climatic variables, such as temperature, humidity, and precipitation.

**Objective.:**

To analyze the relationship between the incidence of dengue and the oceanic Niño index in the departments of the Colombian Caribbean region from 2021 to 2023.

**Materials and methods.:**

An ecological time series study was conducted using distributed lag non-linear models and autoregressive integrated moving average models in the seven departments of the Caribbean region. Descriptive and autoregressive analyses were performed using JASP and RStudio. Non-linear and lagged analyses were run with the dlnmpackage in RStudio.

**Results.:**

A positive and significant relationship between the oceanic Niño index and dengue incidence was found for 2023 data, the year when the El Niño - ENSO (El Niño-Southern Oscillation) warm phase occurred. Bolívar, Cesar, Córdoba, and Magdalena departments showed positive correlations. A non-linear relationship between El Niño/La Niña and dengue incidence was also observed, with a higher increase in dengue cases during El Niño events.

**Conclusions.:**

The oceanic Niño index appears to be a useful climatic indicator for monitoring increases in the monthly dengue incidence rate in the analyzed departments of Colombia’s Caribbean region.

La Organización Mundial de la Salud (OMS) reconoció el cambio climático como una amenaza para la salud pública que impacta a los grupos poblacionales más vulnerables. Este fenómeno tiene un impacto sobre el aumento de la temperatura, la precipitación y la humedad que, a su vez, contribuye al aumento de los casos de dengue, por el virus transmitido por el mosquito *Aedes aegypti*^1^^,^^2^. Específicamente, los eventos de la oscilación del sur de El Niño (*El Niño-Southern Oscillation*, ENSO) también generan un impacto sobre las diferentes variables climáticas y la incidencia de dengue ^3^^-^^5^.

El informe de *Lancet Countdown* 2023 para Latinoamérica resaltó que, en el 2022, la temperatura fue, en promedio, de 0,38 °C más alta que en los años anteriores y, además, destacó que, entre el 2013 y el 2022, los lactantes y las personas mayores de 65 años estuvieron expuestos a un 248 y un 271 % más de días de olas de calor respecto a lo reportado para periodos anteriores. En dicho informe se analizó, también, cómo estos cambios en los ecosistemas -derivados del aumento de las temperaturas- han generado un aumento potencial de la transmisión de dengue en un 54 % para el periodo analizado ^6^.

Varios trabajos han evidenciado la relación entre las variables consideradas como microclimáticas, como la temperatura, la precipitación y la humedad, y los casos de dengue en Colombia. Los investigadores han concluido que las condiciones socioeconómicas se suman a las variables climatológicas para explicar su aumento ^7^^,^^8^. Otros trabajos se enfocaron en la relación entre las variables climatológicas y los casos de dengue para la región Caribe colombiana -específicamente en Santa Marta, Cartagena y Córdoba- y se observó una asociación significativa entre la incidencia de esta virosis y variables como la humedad, la precipitación o el aumento de la temperatura ^9^^-^^11^.

El fenómeno Niño-oscilación del sur, que se considera una variable macroclimática, hace referencia a los patrones de variación en la temperatura de la superficie del océano Pacífico. La ENSO tiene tres fases principales: el Niño, La Niña y la fase neutral; estas fases forman parte de un ciclo natural que se presenta cada dos a siete años. El fenómeno de El Niño contempla eventos climáticos que afectan el aumento de la temperatura de la superficie del océano Pacífico colombiano, lo cual deriva en un aumento generalizado de la temperatura en el territorio nacional. El índice oceánico de El Niño (*Oceanic Niño Index*, ONI) es el indicador que monitorea las fases de la ENSO mediante el cual, a comienzos de julio del 2023, la Organización Meteorológica Mundial informó el inicio de la temporada de El Niño; en Colombia, se oficializó el inicio de dicha temporada en noviembre del 2023 y el Instituto de Hidrología, Meteorología y Estudios Ambientales (IDEAM), según la *National Oceanic and Atmospheric Administration* (NOAA), comunicó su finalización en julio del 2024 ^12^^-^^16^.

La plataforma de información en salud para las Américas (*Health Information Platform for the Americas*, PLISA), respaldada por la Organización Panamericana de la Salud (OPS), informó que en el 2023 se reportaron 4’594.823 casos de dengue en la región de las Américas junto con el fenómeno de El Niño ^17^. En diversos trabajos se ha evidenciado la influencia de El Niño en el aumento de los casos de dengue, a partir de los cuales se ha generado la propuesta de utilizar datos de la ENSO para construir una herramienta de alerta temprana y, así, acelerar la preparación frente a posibles brotes de dengue ^18^^-^^20^.

El Plan Decenal de Salud Pública 2022-2031, resolución 2367 del 2023, presenta siete ejes estratégicos; entre ellos, se destaca el número cinco, denominado “Cambio climático, emergencias, desastres y pandemias”. En este eje, se enfatiza el trabajo intersectorial para la respectiva gestión integral del riesgo frente a un fenómeno complejo como el cambio climático; propone generar conocimiento para abordar el impacto del cambio climático en la salud pública y orientar la formulación de políticas sanitarias. Igualmente, como meta estratégica se espera que, en el 2031, los departamentos y distritos hayan establecido sistemas de alerta temprana para identificar el riesgo en la salud por el impacto del cambio climático y reducir sus efectos en los grupos poblacionales más vulnerables con un enfoque territorial ^21^.

El objetivo del presente trabajo fue analizar la relación entre la tasa de incidencia de dengue en los departamentos de la región Caribe colombiana y el ONI entre el 2021 y el 2023, con el fin de seguir aportando evidencia que permita considerar este índice como una variable del sistema de alerta temprana para la preparación y respuesta ante brotes de dengue a nivel territorial.

## Materiales y métodos

Se desarrolló un estudio ecológico en el que se aplicaron análisis de series de tiempo mediante el uso del modelo de regresión no lineal con desfase temporal (DLNM) y el modelo autorregresivo integrado de promedio móvil (ARIMA) para los datos del 2021 al 2023 de los siete departamentos de la región Caribe colombiana -La Guajira, Magdalena, Atlántico, Cesar, Bolívar, Sucre y Córdoba- para analizar el comportamiento de la incidencia de dengue y su relación con el ONI.

Los datos de dengue se obtuvieron del Sistema de Vigilancia de Salud Pública del Instituto Nacional de Salud (SIVIGILA) del 2021 al 2023. Se seleccionó el periodo mencionado porque permitía analizar la incidencia del dengue durante todo el ciclo ENSO (La Niña-Neutro-El Niño). No se incluyó el 2024 ya que, en el momento de la búsqueda, no se contaba con los datos completos para ese año. Se descargaron las bases completas del país y se filtraron los casos de dengue para cada uno de los departamentos de la región Caribe colombiana. Según la fecha de notificación, se agruparon los casos por mes para compararlos con los datos del ONI, que se reportan mensualmente.

Los datos del ONI se obtuvieron del *Climate Prediction Center-National Oceanic and Atmospheric Administration* (CPC-NOAA) y se reportan en grados Celsius; valores iguales o superiores a 0,5 °C corresponden a la fase de El Niño, valores iguales o inferiores a -0,5 °C hacen referencia a la fase de La Niña, y valores entre 0,4 y -0,4 °C corresponden a la fase neutra.

Con los casos mensuales de dengue, se calcularon las tasas de incidencia por 100.000 habitantes para comparar los departamentos. Con la tasa de incidencia mensual, se generaron análisis estadísticos descriptivos por departamento y por año, y se representaron mediante *boxplots*. Posteriormente, se calculó la tasa mensual de dengue para la región Caribe.

Se evaluó la normalidad de la tasa mensual de dengue mediante la prueba de Kolmogorov-Smirnov; sin embargo, los resultados rechazaron la hipótesis nula de normalidad. Por esta razón, se decidió hacer la correlación entre el dengue y el ONI con la prueba de Spearman (ρ).

La relación entre variables climatológicas y su efecto sobre la incidencia de dengue no es lineal. Esta relación se puede explorar mediante modelos no lineales con desfases temporales ^22^. Se aplicaron los modelos ARIMA y DLNM. Se hicieron análisis estadísticos con el modelo ARIMA para observar las tendencias y el efecto del desfase temporal. El modelo ARIMA comprende tres componentes principales: autorregresivo (AR), integrativo (I) y promedio móvil (MA). Este modelo es el más apropiado para las series de tiempo, ya que permite el análisis histórico, tiene mejor ajuste y solidez (*robustness*) al analizar los patrones dependientes del clima ^23^^,^^24^, y explora relaciones asociativas entre las variables dependientes y las independientes. El modelo ARIMA ha sido eficaz en el análisis de las epidemias de dengue ^25^^,^^26^.

El modelo no lineal con desfase temporal (DLNM) es una herramienta fuerte (*robust*) que permite analizar simultáneamente los efectos no lineales y los retardos o desfases temporales entre los valores del ONI y la incidencia de dengue. La función cross-basis genera una relación bidimensional entre las variables analizadas y los desfases temporales.

Para los análisis descriptivos y de correlación, se utilizó JASP, versión 0.19.3, y, para las asociaciones y desfases, se usaron RStudio, versión 2025.05.0 (Mariposa Orchid) y el paquete dlnm, versión 2.4.10 ^22^^,^^27^. Los gráficos fueron generados en el *software* de RStudio.

## Resultados

**Figura 1:**
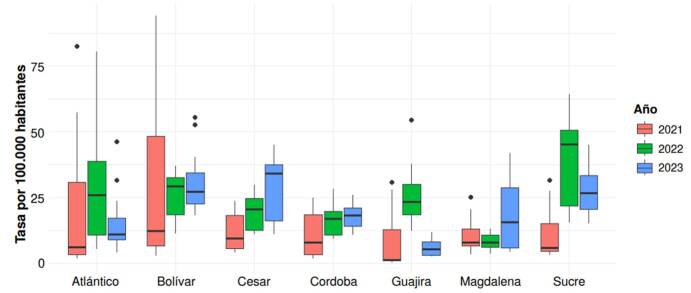
Comparación del número de casos de dengue por 100.000 habitantes entre los departamentos de la región Caribe de Colombia entre el 2021 y el 2023

Con relación a las tasas de dengue, se encontró que en noviembre del 2021 los departamentos de Atlántico y Bolívar presentaron las tasas más altas, de 82,4 y 94,15 por 100.000 habitantes, respectivamente. Para el 2022, las tasas más altas se registraron en el del Atlántico, de 80,5 por 100.000 habitantes en diciembre y, en el de Sucre, de 64,3 por 100.000 habitantes en julio. En el 2023, en el de Bolívar se presentó la tasa más alta (55,5 casos por 100.000 habitantes) junto con el de Atlántico (46,2 por 100.000 habitantes) en enero. El gráfico comparativo anual por departamento se presenta en la figura 1.

**Figura 2:**
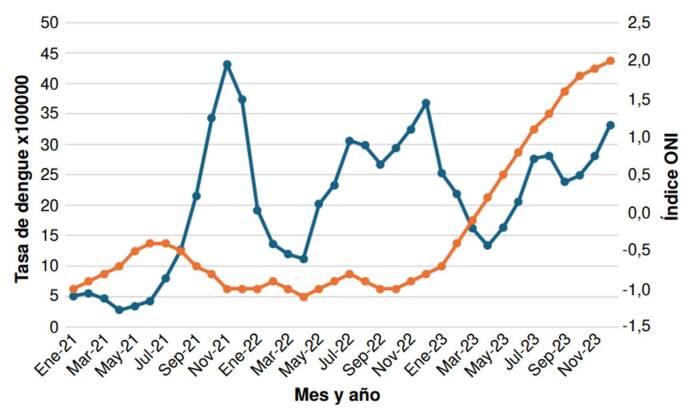
Tasa de casos de dengue por 100.000 (línea azul) para la región Caribe de Colombia y valores del índice oceánico de El Niño (línea naranja) entre el 2021 y el 2023

Entre el 2021 y el 2022, se observaron las mayores tasas mensuales de incidencia de dengue respecto al 2023. En los meses de noviembre y diciembre se presentaron las tasas más altas para cada año. Durante el periodo analizado, predominó La Niña, fluctuando con algunos meses neutros; pero, de mayo a diciembre del 2023, se presentó El Niño (cuadro 1). En el 2023, se observó una tendencia positiva entre la tasa mensual de incidencia de dengue y el fenómeno de El Niño (figura 2).

**Cuadro 1:** Tasa mensual de dengue para la región Caribe de Colombia y valores del índice oceánico de El Niño por año

Mes	Tasa de dengue 2021	ONI 2021	Tasa de dengue 2022	ONI 2022	Tasa de dengue 2023	ONI 2023
Enero	5,1	-1,0	13,7	-1,0	20,8	-0,7
Febrero	5,6	-0,9	12	-0,9	19,1	-0,4
Marzo	4,7	-0,8	11,2	-1,0	15,4	-0,1
Abril	2,8	-0,7	20,2	-1,1	12,3	0,2
Mayo	3,4	-0,5	18,2	-1,0	15,4	0,5
Junio	4,3	-0,4	30,6	-0,9	19	0,8
Julio	7,8	-0,4	29,9	-0,8	22,5	1,1
Agosto	12,7	-0,5	26,7	-0,9	23	1,3
Septiembre	21,5	-0,7	29,4	-1,0	22,1	1,6
Octubre	34,3	-0,8	32,5	-1,0	22,7	1,8
Noviembre	43,2	-1,0	36,9	-0,9	25,2	1,9
Diciembre	29,1	-1,0	36,9	-0,8	29,5	2,0

ONI: índice oceánico de El Niño

La tasa anual de dengue para la región Caribe en relación con el ONI, mostró una relación positiva y significativa (p = 0,778, p = 0,003) solo para el 2023, año en el que se presentó el fenómeno de El Niño. Se obtuvieron correlaciones estadísticamente significativas para las tasas de dengue por departamentos y el ONI por año: en el 2021, se observó una correlación estadística negativa en el departamento del Atlántico; en el 2022, se encontró correlación estadística positiva en los de Bolívar y Magdalena; y en el 2023, se hallaron correlaciones positivas en los de Bolívar, Cesar, Córdoba y Magdalena, mientras que los de La Guajira y Sucre registraron correlaciones negativas (cuadro 2).

**Cuadro 2:** Correlación de Spearman entre la incidencia de dengue y el índice oceánico de El Niño para cada departamento de la región Caribe de Colombia, por año. Se presentan los coeficientes de correlación (ρ) y sus valores de significancia estadística (p).

Departamento	Año	Correlación de Spearman (ρ)	p
Atlántico	2021	-0,585	0,046
Bolívar	2022	0,578	0,049
Bolívar	2023	0,843	< 0,001
Cesar	2023	0,622	0,035
Córdoba	2023	0,830	< 0,001
Guajira	2023	-0,755	0,007
Magdalena	2022	0,678	0,015
Magdalena	2023	0,874	< 0,001
Sucre	2023	-0,755	0,007

Los análisis con modelos no lineales y con desfases temporales presentaron los siguientes resultados: en el análisis del periodo continuo (2021 al 2023), el modelo ARIMA^4^^,^^1^^,^^3^ mostró significancia estadística y un desfase temporal de cuatro meses. Los criterios del modelo ARIMA^4^^,^^1^^,^^3^ fueron: varianza = 666,063, AIC = 10.201,954 y BIC = 10.251,699, los cuales indican que el modelo es aceptable.

**Figura 3:**
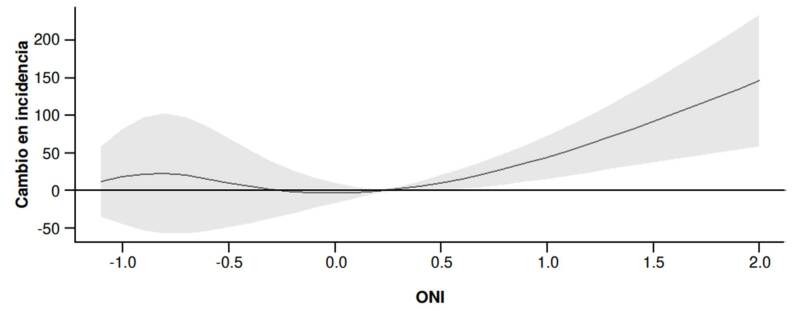
Efecto acumulado del índice oceánico de El Niño sobre el cambio en la incidencia de dengue en la región Caribe de Colombia. La línea negra representa el efecto estimado y la banda gris, el intervalo de confianza del 95 %.

Con el modelo DLNM se generaron dos gráficos: efecto acumulado y efecto bidimensional. En el gráfico con efecto acumulado del ONI sobre la incidencia de dengue, se observa que los valores negativos de este índice durante La Niña se asociaron levemente con la incidencia; mientras que sus valores positivos durante El Niño se asociaron con un aumento progresivo de la incidencia. El cambio de la incidencia de dengue fue mayor durante la fase de El Niño respecto a la de La Niña (figura 3).

**Figura 4:**
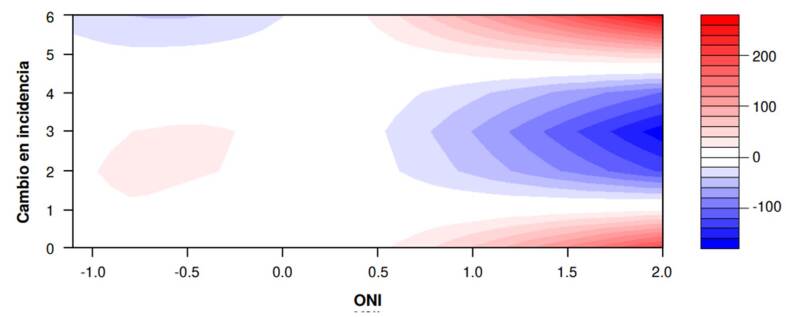
Asociación entre el índice oceánico de El Niño y la incidencia de dengue en la región Caribe de Colombia con diferentes desfases temporales (Lag). La escala de color indica la magnitud y dirección del cambio en la incidencia (rojo: aumento; azul: disminución).

En el gráfico de efecto bidimensional, se observan las variables cruzadas entre los valores del ONI en el eje de las X (La Niña = -0,5 y -1,0; El Niño > 0,5) y el desfase temporal entre 0 y 6 meses en el eje de las Y; el rango de valores a la derecha corresponde al cambio en la incidencia de dengue. Se observa que los valores positivos del ONI en el 2023 se asocian con un aumento de la incidencia de dengue, con un desfase temporal entre 2 y 4 meses; los valores negativos del ONI en el 2021 y el 2022 se asocian con un aumento en la incidencia de dengue, con un desfase temporal entre 5 y 6 meses (figura 4).

## Discusión

El presente trabajo tuvo como objetivo analizar la relación entre la tasa de incidencia de dengue en los departamentos de la región Caribe colombiana y el índice oceánico de El Niño (ONI) entre el 2021 y el 2023. Entre los principales resultados, se resalta que durante el 2021 y el 2022 predominó la fase de La Niña y se presentaron los mayores picos de incidencia de dengue, principalmente en los departamentos de Atlántico, Bolívar y Sucre. En el 2023, con predomino de El Niño, se encontró una relación positiva entre los valores del ONI y el aumento progresivo de la incidencia de dengue. Los análisis no lineales evidenciaron que el cambio de la incidencia de dengue fue mayor durante El Niño comparado con La Niña, con un desfase temporal entre 5 y 6 meses durante La Niña y, de 2 a 4 meses, durante El Niño.

La mayor variabilidad departamental de la tasa de incidencia de dengue durante el 2021 y el 2022 (La Niña) y el aumento progresivo de la tasa de dengue en el 2023 (El Niño) para la región, son hallazgos que confirman que la transmisión del dengue está influenciada por múltiples factores que incluyen, además de los fenómenos climáticos (El Niño o La Niña), las condiciones socioeconómicas, la diversidad geográfica, el entorno, el uso del agua, la infraestructura y el saneamiento, las medidas de salud pública, los aspectos culturales, la urbanización no planeada, la densidad poblacional y la vulnerabilidad social, entre otros ^28^^-^^31^; por lo tanto, es un problema complejo de salud pública.

Con la evidencia científica se ha encontrado que las variables climatológicas afectan los vectores *Aedes aegypti* (vector principal) y *Ae. albopictus* (vector secundario) ya que, a mayor temperatura, se facilita la tasa de replicación del virus del dengue en el vector y se aumenta la frecuencia de la picadura ^28^^,^^32^^,^^33^. A esto se suma que, en una revisión reciente sobre los factores determinantes sociales de la transmisión del virus del dengue, se concluyó que entre los principales factores involucrados se encuentran los ecológicos -incluidos los microclimáticos (temperatura) y los macroclimáticos (el fenómeno de El Niño)-, así como los biológicos (entomológicos y patogénicos) y los sociales a nivel global, comunitario e individual ^34^.

El análisis de los elementos anteriores puede explicar las variaciones de la incidencia del dengue por municipios y departamentos de la región Caribe colombiana, ya que estos han experimentado diferentes procesos de urbanización no planeada y cuentan con microclimas particulares que aportan factores adicionales para el aumento y la dispersión de las enfermedades transmitidas por vectores, como la fiebre de chikunguña y la enfermedad por el virus del Zika ^35^^,^^36^. Los procesos de urbanización de las ciudades del Caribe, caracterizados por la falta de planificación, generan espacios con sistemas de servicios públicos insuficientes e inadecuados ^37^, los cuales propician las condiciones para el aumento de las enfermedades infecciosas transmitidas por vectores, alimentos y roedores ^35^. La relación entre el aumento de la incidencia del dengue y el crecimiento poblacional ha sido documentada en varias ciudades y países como Dhaka City (Bangladesh), Camerún, Río de Janeiro (Brasil), y Guangzhou y Guangdong (China) ^35^^,^^38^^-^^41^.

La tasa de dengue presentó una correlación positiva con el ONI durante la fase de El Niño, lo que sugiere que, a nivel microclimático, el aumento de la precipitación y la temperatura influyen sobre el comportamiento fisiológico del vector que conduce a un aumento de la incidencia de dengue en la región Caribe colombiana. Esto es coherente con lo documentado en un estudio similar en Ecuador para el 2023 y el 2024, en el cual se correlacionó el ONI (fase de El Niño) con el aumento de las enfermedades infecciosas, reportándose un incremento de 13 de ellas ^42^. De manera similar, en Brasil se observó un aumento relacionado con El Niño de las poblaciones de *Ae. aegypti*^32^.

En este trabajo, se evidenciaron la utilidad y la solidez (*robustness*) de los análisis con modelos no lineales para identificar las diferencias en desfases temporales, entre la incidencia de dengue y las fases de La Niña y El Niño. Los resultados del modelo ARIMA permitieron inferir un posible aumento de la incidencia de dengue durante el fenómeno de La Niña con un período de anticipación de cuatro meses, y una relación potencial entre el período de lluvias y el incremento de la proliferación del vector. Este resultado es fundamental para el diseño adecuado de políticas de intervención en salud pública que disminuyan el impacto de la transmisión de dengue en la región Caribe colombiana, mediante acciones anticipatorias ^43^.

El análisis con el modelo DLNM corroboró los datos del modelo ARIMA: evidenció que los efectos de los fenómenos de El Niño y la Niña sobre la incidencia de dengue no son lineales y deben ser analizados con desfases temporales de varios meses, en concordancia con lo sugerido por otros autores ^27^. Se deben hacer futuros análisis con variables macroclimáticas y microclimáticas en períodos continuos y regionales, así como desglosados por año y territorios como lo han sugerido otros estudios ^22^^,^^44^.

Los resultados de los análisis no lineales y con desfases temporales en la relación entre la incidencia de dengue y el ONI, respaldan su utilidad en la generación de sistemas de alerta temprana para dengue en Colombia, propuesta que se plantea como estrategia para fortalecer la gestión de riesgos en cambio climático y salud ^18^^-^^20^. Un sistema de alerta temprana favorece la acción anticipatoria para definir intervenciones preventivas frente a eventos de salud como la fiebre por dengue ^45^. En Perú, se diseñó un modelo que incluyó el ONI como indicador y arrojó pronósticos representativos y confiables de futuros brotes de dengue ^46^. Por lo tanto, un sistema de alerta temprana en el Caribe y en Colombia es necesario y ayudaría a disminuir el impacto sobre las poblaciones vulnerables.

Un análisis previo en diferentes ciudades del Caribe colombiano evidenció que entre el 1972 y el 2017, las ciudades de Barranquilla, Cartagena y Santa Marta presentaron un aumento continuo de la temperatura ^47^; además, según los reportes, hay una relación entre las altas temperaturas y el aumento de los casos de dengue ^48^^-^^50^.

Las limitaciones de este estudio son metodológicas y de alcance. Entre las metodológicas, están las propias de los estudios ecológicos que utilizan fuentes secundarias, por lo cual los análisis dependen de la forma como se recolectan, organizan y publican los datos. Además, al tratarse de análisis grupales, los resultados no permiten establecer relaciones causales, solamente asociaciones entre dos variables sin un elemento explicativo que contemple más aspectos determinantes del problema. Además, no hay un único modelo de análisis de desfases, lo cual limita la comparabilidad de los resultados con otros estudios.

En cuanto a las limitaciones de alcance, en este trabajo no se analizaron variables climatológicas -como la temperatura, la humedad y la precipitación-, ni eventos climatológicos como las olas de calor, que pueden haber influido en los comportamientos diferenciales por períodos o niveles espaciales (departamental o municipal) y que se han relacionado con el aumento del riesgo de transmisión del dengue ^49^. Además, se reconocen otros factores determinantes que no fueron objeto de estudio en este trabajo.

Entre las fortalezas, se destaca el uso de modelos no lineales y con desfases temporales como una herramienta útil para la generación de sistemas de alerta temprana que incluyan el ONI. Este trabajo permitió evidenciar desfases temporales diferentes en las fases de La Niña y El Niño, a partir de lo cual se convoca a la comunidad científica para apoyar el diseño de sistemas de alerta temprana que integren variables climáticas y factores determinantes sociales en las estrategias de salud pública para mitigar el impacto del dengue en contextos vulnerables ^18^^,^^34^^,^^51^. Lo anterior se alinea con los objetivos del Plan Decenal de Salud Pública 2022-2031, que busca la gestión integral del riesgo frente a un fenómeno complejo como el cambio climático. En este caso, la armonización del ONI con los datos del SIVIGILA permite generar modelos predictivos que pueden servir de base para la generación de un sistema de alerta temprana que cuente con la participación de las comunidades.

En conclusión, este trabajo confirma la relación entre el ONI y la incidencia de dengue en la región Caribe colombiana durante el 2021 y el 2023. El Niño se asoció con un aumento más rápido de casos (desfase de 2 a 4 meses) y La Niña presentó un efecto retardado (5 a 6 meses). Los modelos ARIMA^4^^,^^1^^,^^3^ y DLNM confirmaron la utilidad del ONI como predictor no lineal, destacando su potencial para los sistemas de alerta temprana.

## References

[1] World Health Organization Climate change. Key facts.

[2] Abbasi E. (2025). The impact of climate change on travel-related vector-borne diseases: A case study on dengue virus transmission. Travel Med Infect Dis.

[3] Muñoz E, Poveda G, Arbeláez MP, Vélez ID (2021). Spatio-temporal dynamics of dengue in Colombia in relation to the combined effects of local climate and ENSO. Acta Trop.

[4] Dostal T, Meisner J, Munayco C, García PJ, Cárcamo C, Pérez Lu JE (2022). The effect of weather and climate on dengue outbreak risk in Perú, 2000-2018: A time-series analysis. PLoS Negl Trop Dis.

[5] Barrera R, Acevedo V, Amador M, Marzan M, Adams LE, Paz-Bailey G. (2023). El Niño Southern Oscillation (ENSO) effects on local weather, arboviral diseases, and dynamics of managed and unmanaged populations of Aedes aegypti (Diptera: Culicidae) in Puerto Rico. J Med Entomol.

[6] Hartinger SM, Palmeiro-Silva YK, Llerena-Cayo C, Blanco-Villafuerte L, Escobar LE, Diaz A (2024). The 2023 Latin America report of the Lancet Countdown on health and climate change: The imperative for health-centered climate-resilient development. Lancet Reg Health Am.

[7] Rodríguez-Morales AJ, López-Medina E, Arboleda I, Cardona-Ospina JA, Castellanos JE, Faccini-Martínez ÁA (2025). The epidemiological impact of dengue in Colombia: A systematic review. Am J Trop Med Hyg.

[8] Gastelbondo-Pastrana B, Echeverri-De la Hoz D, Sánchez L, García Y, Espitia-Delgado Y, Lopez Y (2024). Climatic variables and their relationship with vector-borne disease cases in Colombia, 2011-2021. Front Trop Dis.

[9] Salazar-Ceballos A, Álvarez-Miño L. (2014). Asociación entre factores climatológicos y tasa de incidencia del dengue en Santa Marta, Colombia, 2007-2013. Rev Cienc Biomed.

[10] Cano-Pérez E, Loyola S, Malambo-García D, Gómez-Camargo D. (2022). Climatic factors and the incidence of dengue in Cartagena, Colombian Caribbean Region. Rev Soc Bras Med Trop.

[11] Medina E, Cogollo MR, González-Parra G. (2024). Prescriptive temporal modeling approach using climate variables to forecast dengue incidence in Córdoba, Colombia. Math Biosci Eng.

[12] McGregor GR, Ebi K. (2018). El Niño Southern Oscillation (ENSO) and health: An overview for climate and health researchers. Atmosphere (Basel).

[13] National Oceanic and Atmospheric Administration Cold and warm episodes by season.

[14] National Center for Environmental Information El Niño-Southern Oscillation (ENSO) 2025.

[15] Ministerio de Ambiente y Desarrollo Sostenible Gobierno nacional declara oficialmente el fenómeno de El Niño y alerta a continuar preparándose.

[16] Instituto de Hidrología, Meteorología y Estudios Ambientales Comunicado Especial N° 073 - Finalización oficial del fenómeno de El Niño.

[17] Pan American Health Organization Plataforma de Información en Salud para las Américas - PLISA. Casos de dengue por país. 2025.

[18] Pramanik M, Singh P, Kumar G, Ojha VP, Dhiman RC. (2020). El Niño Southern Oscillation as an early warning tool for dengue outbreak in India. BMC Public Health.

[19] Andhikaputra G, Lin Y-H, Wang Y-C. (2023). Effects of temperature, rainfall, and El Niño Southern Oscillations on dengue-like-illness incidence in Solomon Islands. BMC Infect Dis.

[20] Ferreira HDS, Nóbrega RS, Brito PV da S, Farias JP, Amorim JH, Moreira EBM (2022). Impacts of El Niño-Southern Oscillation on the dengue transmission dynamics in the Metropolitan Region of Recife, Brazil. Rev Soc Bras Med Trop.

[21] Ministerio de Salud y Protección Social (2023). Resolución número 2367 de 2023 (27 de junio de 2023). Por la cual se adopta el lineamiento técnico para la gestión integral de residuos generados en la atención en salud y otras actividades. Bogotá: Ministerio de Salud y Protección Social.

[22] Gui H, Gwee S, Koh J, Pang J. (2021). Weather factors associated with reduced risk of dengue transmission in an urbanized tropical city. Int J Environ Res Public Health.

[23] Chen X, Moraga P. (2025). Assessing dengue forecasting methods: A comparative study of statistical models and machine learning techniques in Rio de Janeiro, Brazil. Trop Med Health.

[24] Eastin MD, Delmelle E, Casas I, Wexler J, Self C. (2014). Intra- and interseasonal autoregressive prediction of dengue outbreaks using local weather and regional climate for a tropical environment in Colombia. Am J Trop Med Hyg.

[25] Hasan P, Khan TD, Alam I, Haque ME. (2024). Dengue in tomorrow: Predictive insights from ARIMA and SARIMA models in Bangladesh: A time series analysis. Health Sci Rep.

[26] Abdullah NAMH, Dom NC, Salleh SA, Salim H, Precha N. (2022). The association between dengue case and climate: A systematic review and meta-analysis. One Health.

[27] Ortega-Lenis D, Arango-Londoño D, Hernández F, Moraga P. (2024). Effects of climate variability on the spatio-temporal distribution of dengue in Valle del Cauca, Colombia, from 2001 to 2019. PLoS ONE.

[28] Gutiérrez-Barbosa H, Medina-Moreno S, Zapata JC, Chua JV. (2020). Dengue infections in Colombia: Epidemiological trends of a hyperendemic country. Trop Med Infect Dis.

[29] Balaji D, Saravanabavan V, Katturajan K. (2024). Geo-modeling approach of determinants of Chikungunya and its spatial distribution pattern in Madurai city, Tamil Nadu, India. GeoJournal.

[30] Da Conceição Araújo D, Dos Santos AD, Lima SVMA, Vaez AC, Cunha JO, Conceição Gomes Machado de Araújo K. (2020). Determining the association between dengue and social inequality factors in Northeastern Brazil: A spatial modelling. Geospat Health.

[31] do Carmo RF, Silva JVJ, Pastor AF, de Souza CDF. (2020). Spatiotemporal dynamics, risk areas, and social determinants of dengue in Northeastern Brazil, 2014-2017: An ecological study. Infect Dis Poverty.

[32] Pirani M, Lorenz C, de Azevedo TS, Barbosa GL, Blangiardo M, Chiaravalloti-Neto F. (2024). Effects of the El Niño-Southern Oscillation and seasonal weather conditions on Aedes aegypti infestation in the State of São Paulo (Brazil): A Bayesian spatio-temporal study. PLoS Negl Trop Dis.

[33] Nik Abdull Halim NMH, Che Dom N, Dapari R, Salim H, Precha N. (2022). A systematic review and meta-analysis of the effects of temperature on the development and survival of the Aedes mosquito. Front Public Health.

[34] Barkhad A, Lecours N, Stevens-Uninsky M, Mbuagbaw L. (2025). The ecological, biological, and social determinants of dengue epidemiology in Latin America and the Caribbean: A scoping review of the literature. Ecohealth.

[35] Kolimenakis A, Heinz S, Wilson ML, Winkler V, Yakob L, Michaelakis A (2021). The role of urbanization in the spread of Aedes mosquitoes and the diseases they transmit-A systematic review. PLoS Negl Trop Dis.

[36] Baker RE, Mahmud AS, Miller IF, Rajeev M, Rasambainarivo F, Rice BL (2021). Infectious disease in an era of global change. Nat Rev Microbiol.

[37] Ricardo-Rivera SM, Aldana-Carrasco LM, Lozada-Martínez ID, Bolaño-Romero MP, Acevedo-Lopez N, Sajona-Leguia WA (2022). Mapping dengue in children in a Colombian Caribbean Region: Clinical and epidemiological analysis of more than 3,500 cases. Infez Med.

[38] Ritu MR, Sikder D, Patwary MM, Tamim AR, Rodríguez-Morales AJ. (2024). Climate change, urbanization and resurgence of dengue in Bangladesh. New Microbes New Infect.

[39] Kamal ASMM, Al-Montakim MN, Hasan MA, Mitu MMP, Gazi MY, Uddin MM (2023). Relationship between urban environmental components and dengue prevalence in Dhaka city -An approach of spatial analysis of satellite remote sensing, hydro-climatic, and census dengue data. Int J Environ Res Public Health.

[40] Montgomery MJ, Harwood JF, Yougang AP, Wilson-Bahun TA, Tedjou AN, Keumeni CR (2025). The effects of urbanization, temperature, and rainfall on Aedes aegypti and Aedes albopictus mosquito abundance across a broad latitudinal gradient in Central Africa. Parasit Vectors.

[41] Pereira PAS, Martins ACCT, Souza ER de O, Pontes AN. (2020). Perfil epidemiológico da dengue em um município do norte brasileiro: uma análise retrospectiva. Res Soc Dev.

[42] Molleda P, Velásquez Serra G. (2024). El Fenómeno del Niño y la prevalencia de enfermedades infecciosas: revisión. Granja.

[43] Lin C-H, Wen T-H. (2024). Assessing the impact of emergency measures in varied population density areas during a large dengue outbreak. Heliyon.

[44] Sugeno M, Kawazu EC, Kim H, Banouvong V, Pehlivan N, Gilfillan D (2023). Association between environmental factors and dengue incidence in Lao People’s Democratic Republic: A nationwide time-series study. BMC Public Health.

[45] Glantz MH, Ramírez IJ. (2025). Enhancing societal value of early warning, early action, and anticipatory action frameworks using NOAA’s Oceanic Niño Index. Int J Disaster Risk Sci.

[46] Mills C, Donnelly CA. (2024). Climate-based modelling and forecasting of dengue in three endemic departments of Perú. PLoS Negl Trop Dis.

[47] Salazar-Ceballos A, Álvarez-Miño L. (2020). The heat index: An early warning factor in public health and sustainable cities. Salud Uninorte.

[48] Frentiu FD. (2023). Dengue fever: The impact of increasing temperatures and heatwaves. EBioMedicine.

[49] Damtew YT, Tong M, Varghese BM, Anikeeva O, Hansen A, Dear K (2023). Effects of high temperatures and heatwaves on dengue fever: A systematic review and meta-analysis. EBioMedicine.

[50] Cheng J, Bambrick H, Yakob Laith, Devine G, Frentiu FD, Thanh T (2020). Heatwaves and dengue outbreaks in Hanoi, Vietnam: New evidence on early warning. PLoS Negl Trop Dis.

[51] Ortiz-Prado E, Camacho-Vasconez A, Izquierdo-Condoy JS, Bambaren C, Hernández-Galindo L, Sanchez JC. (2023). El Niño-Southern Oscillation: A call to action for public health emergency preparedness and response. Lancet Reg Health Am.

